# Traditional Chinese medicine for irritable bowel syndrome: from classic formula to modern medicine development

**DOI:** 10.1186/1479-5876-10-S2-A35

**Published:** 2012-10-17

**Authors:** Zhaoxiang Bian

**Affiliations:** 1 School of Chinese Medicine, Hong Kong Baptist University, Hong Kong, China

## Background

Irritable bowel syndrome (IBS) is a very common functional gastrointestinal disease. Epidemiological studies showed that 14% of males and 27% of females in the US (white) have symptoms of IBS. In Hong Kong, one survey reported a similar prevalence of this condition among Chinese (3.6% in males and 3.8% in females with Rome II criteria). Because the mechanism of IBS is not well understood, the IBS sufferers are not satisfactory about the symptoms management; and the new chemical drug development targeting IBS is facing big challenges. In traditional Chinese medicine, there is no term of IBS, although many classic literature record coving the pathophyioloigal concepts and therapeutic approaches bout “painful diarrhea”.

## Results

Our research group have developed a new Chinese medicine formula, called JCM1602, based on “Important Formula for Painful Diarrhea” and our clinical therapeutic experience. The aim of our study is to develop a modern new drug based on the Chinese medicine theories and modern pharmaceutical approaches. For this purpose, firstly, we evaluated the efficacy and safety of JCM16021 in a randomized double blinded, double dummy, control study. Eight four patients were assigned to JCM1602 plus placebo holopon arm, holopon plus placebo JCM1602 arm and double placebo arm; and all patients went through 8 weeks treatment and 8 weeks follow-up. The primary outcome is the general improvement of symptoms. Results showed that IBS patients with JCM1602 have significant symptom relieve during the treatment period and the follow up period, comparing with that of the holopon group and placebo group. Based on the clinical study results, a new Chinese medicine drug development process was adapted from the pharmacognosy, pharmacology, efficacy, safety aspects. Further, two animal models, including neonatal maternal separation-induced visceral hyperalgesia model, 2,4,6-trinitrobenzene sulfonic acid-induced post inflammatory IBS model, have been applied for the efficacy confirmation and mechanism discovery.

## Conclusion

The results support the efficacy of JCM1602 in patients, and found that the analgesic effect and anti-diarrhea effect is accomplished through serotonin pathway. Furthermore, the active fraction of JCM1602 was identified. This active fraction is the base for the new drug development for IBS.

